# Primary cilium: a paradigm for integrating mathematical modeling with experiments and numerical simulations in mechanobiology

**DOI:** 10.3934/mbe.2021066

**Published:** 2021-01-15

**Authors:** Zhangli Peng, Andrew Resnick, Y.-N. Young

**Affiliations:** 1 Department of Bioengineering, University of Illinois at Chicago, 851 S. Morgan St., Chicago, IL 60607, USA; 2 Department of Physics, Center for Gene Regulation in Health and Disease, Cleveland State University, Cleveland, OH 44115, USA; 3 Department of Mathematical Sciences, New Jersey Institute of Technology, University Heights, Newark, NJ 07102, USA

**Keywords:** cilium, fluid-structure interactions, elastohydrodynamics, mechanosensing, slender-body

## Abstract

Primary cilia are non-motile, solitary (one per cell) microtubule-based organelles that emerge from the mother centriole after cells have exited the mitotic cycle. Identified as a mechanosensing organelle that responds to both mechanical and chemical stimuli, the primary cilium provides a fertile ground for integrative investigations of mathematical modeling, numerical simulations, and experiments. Recent experimental findings revealed considerable complexity to the underlying mechanosensory mechanisms that transmit extracellular stimuli to intracellular signaling many of which include primary cilia. In this invited review, we provide a brief survey of experimental findings on primary cilia and how these results lead to various mathematical models of the mechanics of the primary cilium bent under an external forcing such as a fluid flow or a trap. Mathematical modeling of the primary cilium as a fluid-structure interaction problem highlights the importance of basal anchorage and the anisotropic moduli of the microtubules. As theoretical modeling and numerical simulations progress, along with improved state-of-the-art experiments on primary cilia, we hope that details of ciliary regulated mechano-chemical signaling dynamics in cellular physiology will be understood in the near future.

## 1. Introduction

### 1.1. Primary cilia are the most uncomplicated members of the ‘cilia family’

Primary cilia are the least adorned version of a subcellular organelle common to most eukaryotic cells and protozoa. Here ‘cilia’ and ‘flagella’ refer to the same object, their names were given prior to microscopic structural observations, and we refer to the collected family of ciliary and flagellar structures as ‘cilia’. All cilia share the same basic protein structure [1], consisting of nine axonemal microtubule doublets (encased by the ciliary membrane) that extend outward from centriolar microtubule triplets.

Cilia are to be distinguished from bacterial pili, bacterial flagella, eukaryotic microvilli, and epithelial brush border and glycocalyx. These structures do not consist of microtubules and do not have the same functions as cilia.

Primary cilia have the minimal structure within the family of cilia. While all cilia have the same essential conserved structures, primary cilia lack a central microtubule pair and associated motor proteins. Consequently primary cilia do not actively move (‘beat’) and can be modeled as passive structures. In contrast, nodal cilia are a member of the cilia family that has the same axonemal structure as primary cilia but in addition, can rotationally move in a ‘twirling’ motion. Nodal cilia only appear early and briefly in embryo development and are required to establish left-right asymmetry [2].

Models of motile and nodal cilia are primarily concerned with the molecular mechanisms and regulation of beating rather than modeling a mechanosensory function. However, because cilia structure is conserved, we expect that mechanotransduction models of primary cilia will be applicable to most the cilia family.

Primary cilia are also the minimal subcellular structure that convincingly displays flow-sensing. Many cell types integrate primary cilia into larger sensory apparatuses: hair cells, for example, perform their sensing function with a primary cilium (‘kinocilium’) that is attached to a bundle of actin filaments (confusingly named ‘stereocilia’), all connected by extracellular protein links (‘tip links’). A second example is focal adhesion remodeling: a common response of many adherent cells to an applied basolateral shear force [3]. This mechanotransduction response is fairly well studied [4] but the relevant biology and physiology are not clearly related to ciliary sensing [5]: the relevant signaling pathways and responses are completely disjoint from apical fluid flow sensing.

### 1.2. Anatomy of primary cilium

**Figure 1.**
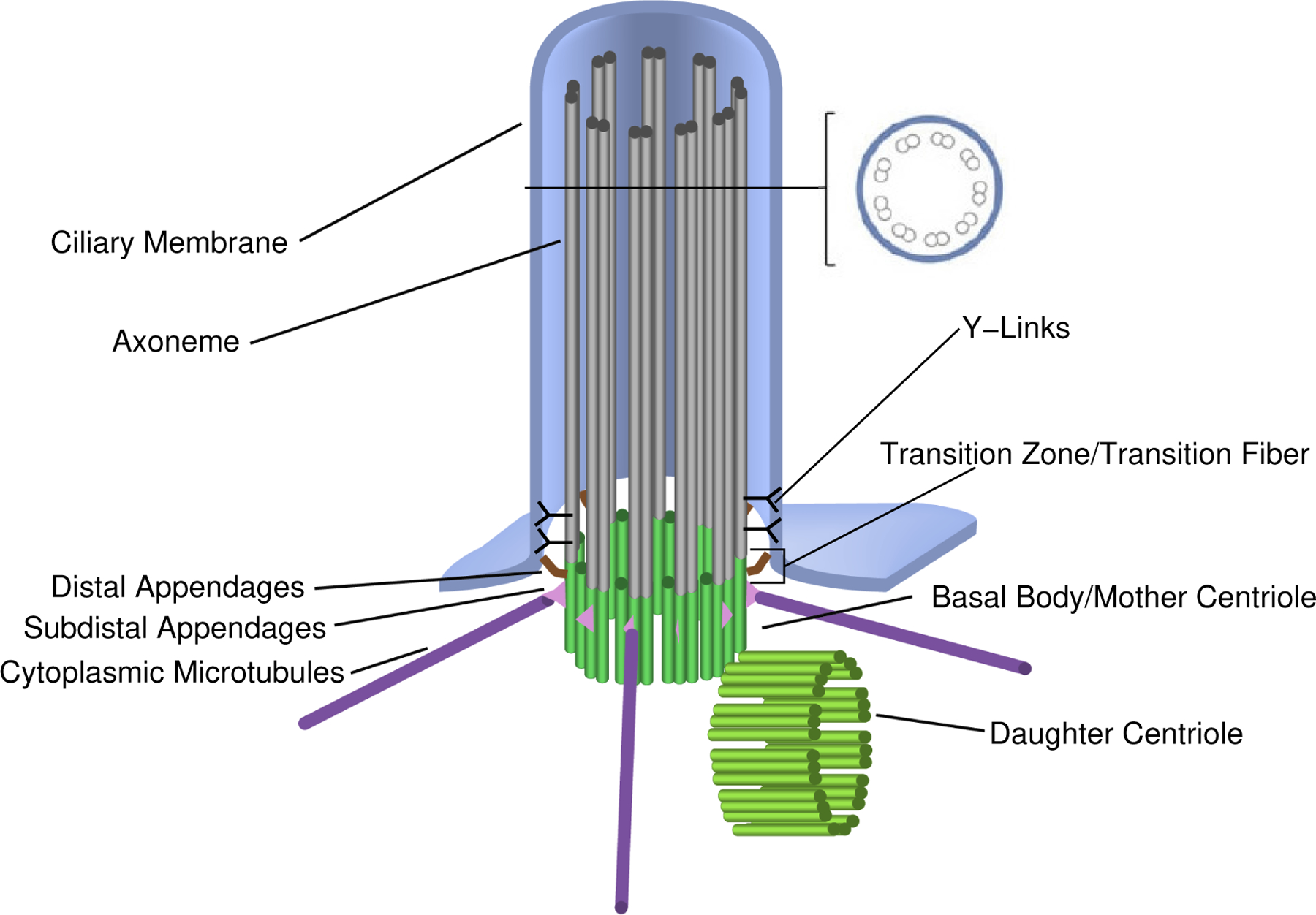
Schematic diagram of primary cilium (adopted from Temiyasathi et al. [14])

A sketch of a primary cilium with some details of the microtubule (MT) arrangements is shown in Figure 1, where a ciliary membrane (extended from the plasma membrane [6]) encloses the nine MT doublets [7, 8] with several structures around the axonemal base. The basal centrosome disk is of 200 nm in diameter, and the length of the ciliary axoneme is in the range of 1~10 *μ*m.

Experimental findings suggest that the ciliary membranes are physiologically different from the plasma membrane in lipid compositions [9, 10], electrical resistance [11], ion-binding, and osmotic behavior [12, 13]. The axonemal base is connected to the cilium basal body, a protein-based structure consisting of nine microtubule triplets arranged circumferentially.

Primary cilia differ from the better-understood motile cilia in several important aspects. Unlike motile cilia, there is only a single primary cilium per cell. Primary cilia are found in virtually every mammalian cell type, while motile cilia are mostly expressed in specialized cells. Primary cilia have multiple basal feet and striated rootlets distributed evenly around their basal bodies, while the basal feet of beating cilia are often aligned with the beating direction [15].

Furthermore motile cilia and flagella have two additional central MTs, which are attached to each of the surrounding doublets by radial spokes. Therefore motile cilia are commonly referred to as 9 + 2 cilia in contrast to the 9 + 0 primary cilia. In addition, the doublets of the 9 + 2 cilium are connected via nexin links, which (in combination with the radial spokes) reinforce the axoneme rigidity, resulting in an order of magnitude larger resistance to bending (flexural rigidity) relative to the primary cilium.

For both motile and primary cilia the axonemal structure and length regulation converge on the status of the ciliary tip. While the ciliary tip is thought to be the site of ciliary length regulation, microtubule stability, and overall maintenance of the cilium, the distal end of a cilium remains poorly characterized. The primary reason is the difficulty of locating a cilium tip in thin-sectioned material suitable for electron microscopy. A good review of various proposed ciliary tip models is presented in [16]. Ciliary tip structure has additional significance for airway motile cilia, as short actin appendages have been proposed to couple ciliary motion with mucus transport [17].

At the proximal end, the axoneme terminates into another highly organized protein complex, the so-called ‘transition zone’. This region is a complex structure that (I) brings together the axonemal microtubule doublets with centrosomal microtubule triplets and (II) connects the ciliary membrane with the cell apical membrane. While the protein content of this region has not yet been fully determined, it is suspected that the transition zone in part acts similar to a molecular sieve or pore complex regulating the transport of material into and out of the ciliary compartment.

Various subcompartments of cilia are reviewed in [6, 18–22]. The functions of these subcompartments are beginning to be understood. For example, the basal end of the cilium forms a size-exclusion diffusion barrier (the ‘ciliary gate’) with a permissive size cuto of 7.9 nm [23], similar to nuclear pore complexes [24]. In addition to the selective gated transport of material into and out of the cilium, it is also possible that the ciliary gate is used to distinguish between mechanical and chemical stimulus [25]. Examining the role of the basal body, Espinha et al. [26] demonstrated that applied oscillatory flow increases the number of cytoskeletal microtubules attached to the basal body, potentially identifying an adaptive response via modification of the ciliary ‘anchor’.

While contiguous with the plasma membrane, the ciliary membrane has a different lipid composition [27]. However, the role of the cell membrane (as opposed to ciliary transmembrane proteins) mediating ciliary function has only been partially studied. Several studies [25, 28–30] have demonstrated that the cilioplasm is a distinct subcellular compartment, possibly maintained by the highly structured transition zone.

It has been long-known that cilia could be visualized fluorescently by using standard immunostaining techniques directed against acetylated alpha-tubulin (ciliary axoneme), gamma-tubulin (basal body), and other ciliary-localized proteins. Ciliary proteomics (ciliome) was first established in [10] and has continued to be refined over time. A current database can be found at http://www.sfu.ca/leroux/ciliome_database.htm and referenced in [31]. Information about the ciliome is crucial as hundreds of different proteins are localized to the cilium to support the wide variety of sensing and signaling functions. To take one example, studies (reviewed in [32]) of the post-translational modification of tubulin provide important clues as to the function of tubulin acetylation, namely that acetylation is a marker of microtubule stability as opposed to an active role stabilizing microtubules [33].

Because the diameter of cilia is approximately 200 nm, ciliary axonemes are just barely detected using light microscopy. Most ultrastructural studies of cilia combine light and electron microscopy [34], a comprehensive review is found in [16]. Electron microscopy has been used to image cilia for many decades, and steady improvement of the technique continues to provide new information about the ultrastructure. Immunofluorescence imaging of fixed cilia often uses an anti-body directed against acetylated-tubulin to stain the ciliary axoneme [35] as a positive control. However, it is becoming more appreciated that fixation of samples irreversibly damages cilia [36, 37], and so live cell imaging is preferred. Complicating live-cell microscopic observations is that the cilium is oriented along the optical axis of the microscope; efforts to culture cells such that the cilium grows perpendicularly to the optical axis are periodically published [25,38].

### 1.3. Physiology of primary cilium

Defective or malfunctional primary cilia have been linked to pathologies [39–43] such as arthritis, osteoporosis, polycystic kidney disease, heart failure, obesity and cancer. For example, it is well-known that when bone is exposed to repeated mechanical loading in vivo, it loses its sensitivity to subsequent mechanical loading [44, 45]. This reduced sensitivity is refractory and is gradually reversed over a period of minutes. Furthermore, it coincides with a loss of cellular responsiveness [46]. Interestingly, there is also evidence that primary cilia degrade with loading [47]. Finally, it is important to note that the existence of a primary cilium is predicated on that cell having exited the cell cycle, as the basal body (centrosome) is required during mitosis to separate the two chromosomes. This relationship between the ciliogenesis and cell cycle has itself created a large body of results and raises several fundamental questions in developmental biology, including both cellular de-differentiation and wound healing.

The flow-sensing role of primary cilia was discovered when Praetorious and Spring found a dramatic extracellular calcium-dependent increase in intracellular calcium by using fluid flow or a micropipette to bend the primary cilia of kidney epithelial cells [48]. They also found that this response was lost with removal of primary cilia [48, 49]. Rydholm et al. [50] constructed a three-dimensional elastic model of the cilium incorporating the cell membrane as an additional, independent, structural element and compared their model to experiments.

It has been suggested that the flow sensing response occurs via a cationic channel that localizes to the base of the cilium known as polycystin-2 [51, 52]. This mechanism has also been found in liver cholangiocytes in response to fluid flow [53]. In addition to calcium signaling, fluid flow in bile ducts also activates the second messenger cAMP [53]. The response of the primary cilium to the external load is observed to be more than mechanical bending and transfer of stress, but also molecular alterations. For example, proteins of the linker density may undergo conformational changes as a result of Ca^2+^ binding, which increases the stiffness of stereocilia [54] and also increases the rate of depolymerization of microtubules [55,56].

### 1.4. Modeling of primary cilium

We distinguish between mechanical (structural or anatomical) models of the primary cilium from functional (physiological) models of the primary cilium. Overwhelmingly, existing “mathematical models” of the primary cilium have been mechanical in nature. Functional models of the primary cilium do not yet exist in any substantive detail and represent an emerging area of study.

Motile cilia and flagella are examples [57–68] where, once the biomechanics of an organelle is reasonably well approximated in mathematical terms, advancements in multiscale modeling and direct numerical simulations lead to quantitative understanding of biological functions that may be beyond reach of experiments alone. These advances have also stimulated a series of experiments focusing not just on more detailed investigations of motile cilia, but also synthesis of artificial beating cilia that are designed to perform the biological function of moving mucus [69,70].

Compared to motile cilia and flagella, much less theoretical modeling and numerical simulations of primary cilia have been reported. Schwartz et al. [71] modeled the primary cilium as a small-deformation elastic beam. Resnick et al. [72] applied a similar formulation to study small deflections in cylindrical Poiseuille flow. Liu et al. [73] used a more precise model of the fluid flow around an array of cilia by numerically solving Stokes equations. They assumed small rotation at the cilium base although they computed the drag on cilium axoneme consistently from Stokes equations. Rydholm et al. [50] conducted fluid dynamics simulations of the bending of an elastic filament connected to an elastic membrane. They found that, for an elatic beam bending under flow, the maximum stress is located at the base that is connected to an elastic sheet without anchorage in their simulation setup. They also conducted experiments illustrating the cilium bending and the subsequent variation in the intracellular calcium concentration. To our knowledge the only modeling work that includes the support from the complex ciliary base is by Young et al. [74], who modeled the basal anchorage of the primary cilium as a rotational spring with reasonably good agreement with experiments (see section 3.1).

## 2. Experiments

### 2.1. Biomechanics of primary cilium

In section 1.2 we stated that the aspect ratio (length/radius) of primary cilia is approximately 10 and can exceed 500. Consequently measurements of cilia and flagella [75] were interpreted within the “slender body model”. In this schema, cilia and flagella are characterized in terms of a beam length, basal anchoring, and macroscopic continuous properties of the beam such as Young’s modulus “E” [71].

The basal elastic strain energy of a deflected Bernoulli-Euler cantilevered beam depends on both the elastic bending modulus and length; thus implying that ciliary length control may have a functional relevance in flow sensing. In contrast to a large body of results identifying intracellular biochemical (i.e., independent of external mechanical stimuli) length regulation mechanisms [76], experimental evidence of external mechanical stimulus length regulation mechanisms remains fragmentary: applied flow shortens cilia [72], and chronic obstruction of flow results in increased cilia lengths. In the context of renal injury and recovery, resolution of the obstruction returns cilia to preinjury length [77, 78]. Removal of one kidney results in elongation of cilia in the remaining kidney [79].

The bending modulus (sometimes “flexural rigidity”) EI is a parameter used in elastic beam theory to describe the deformation of a beam in response to an applied external load. An excellent summary of different experimental approaches used to determine EI can be found in [80].

Bending modulus measurements of individual MTs again shows persistent disagreement. Careful experiments [81, 82] on pure MTs resulted in EI varying between 3.7 × 10^−24^ and 35.8 10^−24^ N m^2^. It has been demonstrated that both the average value of EI and the statistical distribution× of EI values can be altered by pharmacological stabilization [83]. The disparities in measured results has recently been resolved by characterizing MTs as orthotropic tubes [84–86].

As a set of parallel results, we have recently characterized the bending modulus of primary cilia [87]. We showed that addition of nanomolar amounts of taxol decrease the bending modulus in agreement with MT bending measurements [88]. In addition, we demonstrated that adding 100 nM of CoCl_2_ [to stabilize hypoxia-inducible factor (HIF)] results in more flexible cilia [87], in agreement with prior results [89] given the hypothesis that cilium length and flexibility are related.

Interestingly, experimental measurements of the deformation of a primary cilium have converged to show that when modeling a cilium as an elastic beam, the bending modulus is only slightly larger than that of a single MT. This apparent paradox has not fully been resolved, but progress has been made by modeling cilia in terms of orthotropic tubes [90], similar to MT models.

A step forward in cilia research occurred with the development of a mouse model, the ORPK mouse model [91]. This engineered mouse has a genetic disruption of the intraflagellar transport apparatus, specifically deletion of gene *ift88*, and as a result both motile and immotile cilia are much shorter than normal, wildtype, mouse cells.

### 2.2. Flow-sensing of primary cilium

Extracellular fluid flow generates shear forces acting directly on the apical plasma membrane and hydrodynamic drag acting on protruding structures such as cilia. Fluid shear can initiate cilia-independent flow responses, for example activation of MAPK signaling and PGE2 secretion. Shear and stretch could potentially generate confounding effects obscuring the cilia-fluid flow response, and it is experimentally challenging to separate cilia-dependent effects from confounding cilia-independent responses.

The ORPK mouse model has facilitated many experiments exploring the mechanistic connection between ciliary bending and intracellular Calcium signaling leading to a focus on the mechanics of protein Polycystin-1 and identification of Polycystin-1/Polycystin-2 complexes (PC-1/PC-2) as main candidates in ciliary mechanosensation [52,92,93]. PC-1 is thought to be the mechanosensor that acts to open PC-2. PC-1 is a large transmembrane protein with a long extracellular ‘tail’ that is cleaved [94]. PC-1 forms complexes with PC-2, a Calcium channel [95]. In addition, there are Polycystin-like proteins (PCL1 and PCL2) also found on cilia but with unknown functions [96].

A variety of signaling molecules regulate ciliary length [55, 89, 97–101], and yet solid connections between ciliary flow-sensing and ciliary length has not yet been established.

## 3. Mathematical modeling

### 3.1. An isotropic elastic beam in a viscous fluid

The aspect ratio (*ϵ*) of a primary cilium, defined as the ratio of cross-sectional radius *r* to length *L* of the center-line, is often in the range 10^−2^ ≤ *ϵ* ≤ 10^−1^. Consequently the primary cilium dynamics under flow can be well-approximated by the bending of an anchored elastic slender filament under a hydrodynamic load [62,64,102–104].

**Figure 2.**
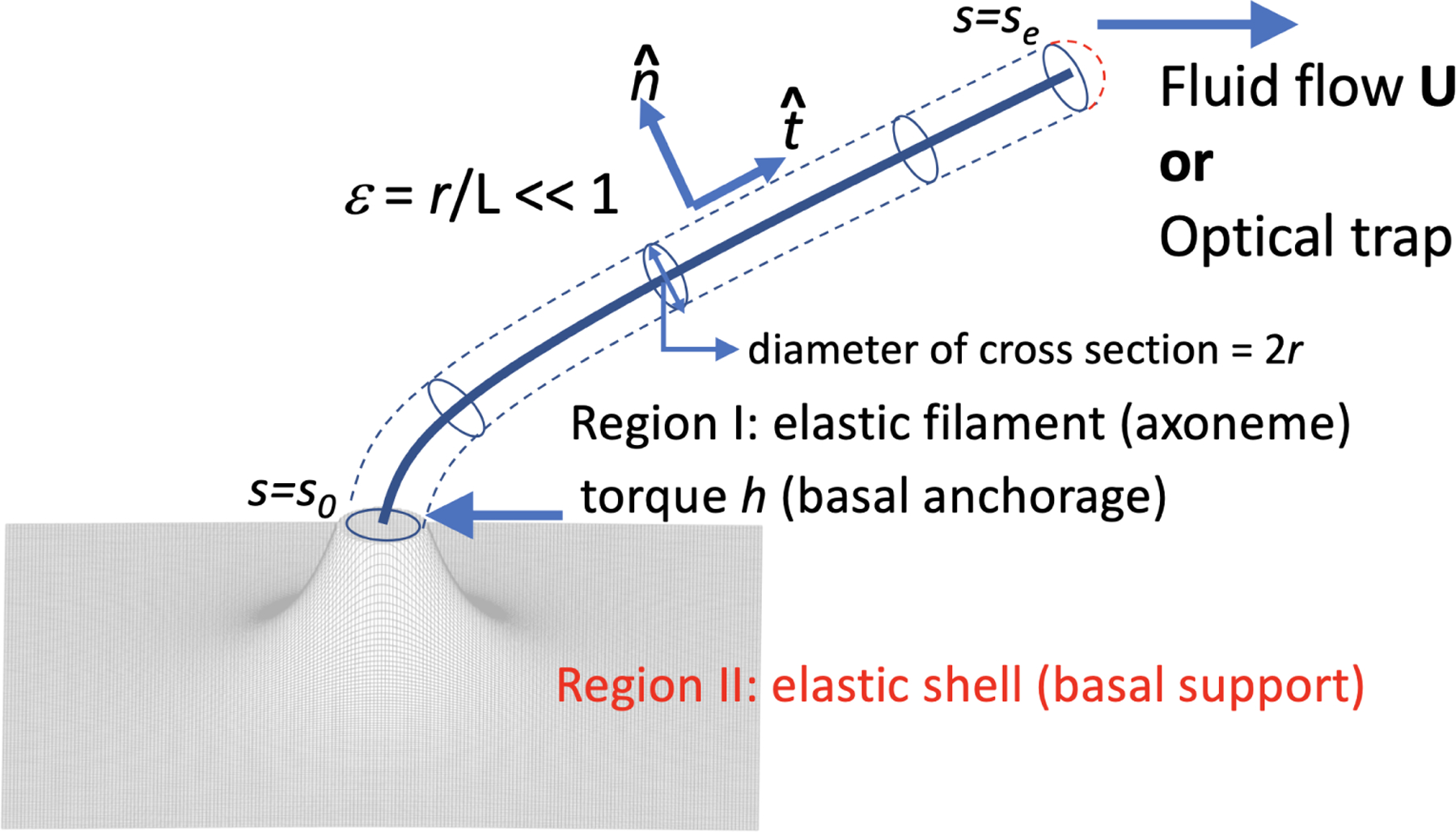
Schematics of the primary cilium as an isotropic elastic beam (region I) in the slender-body theory framework, where a external fluid flow **U** or an optic trap can bend the elastic filament. The basal anchorage of a primary cilium is modeled as the support from coupling to an elastic shell (region II).

In the slender-body framework, the filament is described by a center-line parametrized by arc-length *s* ∈ [*s*_0_, *s*_*e*_], see Figure 2. The force distribution along the slender-body center-line is decomposed in the tangential $\hat{t}$ and the normal $\hat{n}$ directions (as $F\;=\;F^{t}(s)\hat{t}\;+\;F^{n}(s)\hat{n}$) (Figure 2). Assuming linear elasticity for the ciliary axoneme, the curvature *κ* is thus proportional to the moment *M*: *M* = *E*_*B*_*κ*, where *E*_*B*_ is the bending rigidity. The distributed load $P(s)\;=\;P^{t}\hat{t}\;+\;P^{n}\hat{n}$ is related to the force by $\frac{dF}{ds}\;+\;P\;\equiv \;F_{s}\;+\;P\;=\;0$. The moment and the force are related as $\frac{dM}{ds}\;=\;M_{s}\;=\;F^{n}$. Denoting the filament center-line **x** = (*x*(*s*), *y*(*s*)) and $\hat{t}\;=\;\left(t_{1}(s),\;t_{2}(s)\right)$, the governing equations are obtained by force balance for an elastic filament of constant length, see [74] for a more detailed derivation. The hydrodynamic load **P** is computed from the local SBT as

(3.1) $$P\;=\;-\frac{\eta (\partial x/\partial t\;-\;U)}{(1+2\beta )I\;+\;(1\;-\;2\beta )x_{s}\;⊗\;x_{s}},$$

where **x**_*s*_ *≡ d***x**/*ds*, **U** is the fluid velocity at the location **x** in the absence of the elastic filament, *β* = 1/(− ln(*ϵ*^2^*e*)) is the filament slenderness and $\eta \;=\;\frac{8\pi \mu \dot{\gamma }L^{4}\beta }{E_{B}}$ is the effective viscosity with *μ* the fluid viscosity and $\dot{\gamma }$ the characteristic flow rate. The parameter *η* quantifies the magnitude of the viscous force relative to the restoring elastic force. This formulation is also generalized for time-dependent dynamics of an inextensible elastic filament under flow [74,105].

At the free filament end (*s* = *s*_*e*_), the force-free and torque-free conditions give

(3.2) $$F^{t}\left(s_{e}\right)\;=\;0,\;F^{n}\left(s_{e}\right)\;=\;0,\;\kappa \left(s_{e}\right)\;=\;0.$$

At the basal body (*s* = *s*_0_) the filament endpoint is fixed: *x*(*s*_0_) = 0 and *y*(*s*_0_) = 0. Applying the above elastic beam model to match the equilibrium cilium profiles under a steady shear flow, it is found that (a) the bending rigidity of the ciliary axoneme is in the range of 1 ≤ *E*_*B*_ ≤ 5 × 10^−23^ N m^2^, and (b) the basal anchorage behaves as a nonlinear rotational spring, whose spring constants can be found by fitting the elastic filament profile from the SBT to the experimental observations. Using this model (SBT for the elastic filament with a rotational spring at the filament base), Young et al. were able to reproduce the deflection dynamics of a primary cilium under a planar shear flow with good agreement with experiments [74].

To understand the nature of basal anchorage, Young et al. [74] coupled the elastic beam to an elastic shell with a torque (*h*, see Figure 2) at the junction to model the basal anchorage, which is found to behave like a nonlinear rotational spring [74]. Similar approaches have also been adopted in earlier efforts to understand the primary cilium bending mechanics [106,107]. The elastic sheet has a bending rigidity *E*_*T*_ ≡ *λE*_*B*_ with equations λ~O(1) [74]. The interested readers are referred to [74] for the governing of the elastic sheet with axial symmetry. At the junction where the elastic shell is connected to the filament base, the unit tangent vector is reasonably assumed to be continuous from the filament base to the elastic shell [74]. Secondly, the force distribution and the curvature are also assumed to be continuous at the junction.

### 3.2. An anisotropic elastic beam

**Figure 3.**
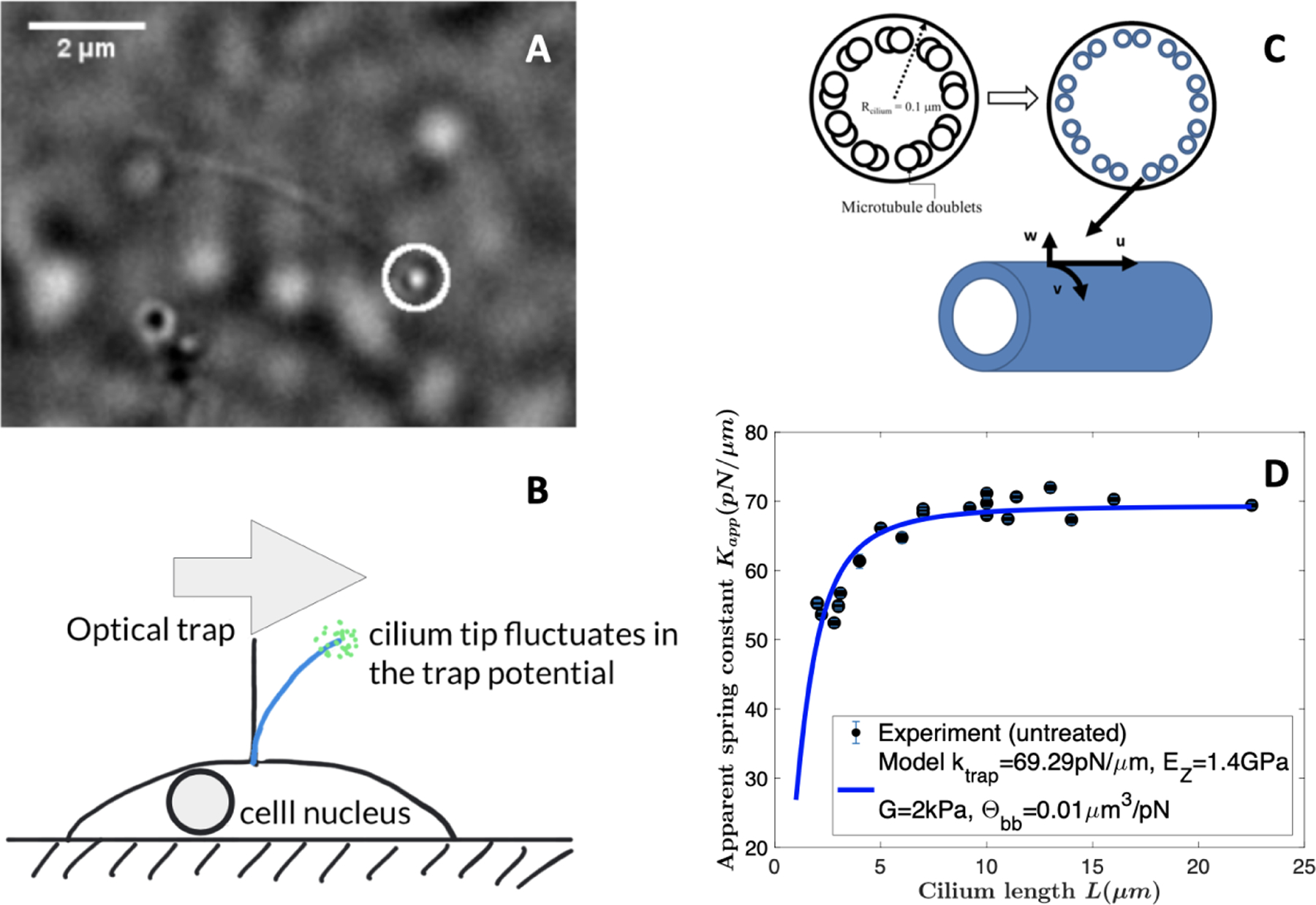
A: Top view of a primary cilium bent by an optical tweezer. B: A schematic showing the fluctuating cilium tip under an optical trap. C: An illustration of an orthotropic elastic shell for the ciliary axoneme. D: Effective spring constant of a primary cilium versus its contour length *L* (for more details see [90]).

When we applied the above model for an isotropic elastic beam to model a primary cilium under an optical trap (Figure 3A,B) and compute the effective ciliary spring constant, we found inconsistency in the scaling of the spring constant with respect to the cilium length between modeling and experiments [90].

Flaherty et al. showed that the primary cilium is better modeled as a transversely isotropic elastic shell [90] (Figure 3C). Focusing on the stochastic dynamics of cilium tip under an optical trap and how such dynamics is coupled to the stochastic displacement of the cilium base, Flaherty et al. concluded that the cilium axoneme has to be modeled as an anisotropic elastic shell in order to interpret the experimental findings on the effective ciliary spring constant in Figure 3D.

It is important to note that these two models cannot be made equivalent; while material properties (Young’s modulus for a homogeneous beam, both Young’s and shear moduli for orthotropic tubes) can be found that result in identical deformation profiles for the two models, this can only occur for a single length. Because the length of a cilium is not a fixed constant, it is not possible in general to fit both models simultaneously to the data. This point is also discussed in [90].

## 4. Numerical simulations

### 4.1. Biomechanics of primary cilium

**Figure 4.**
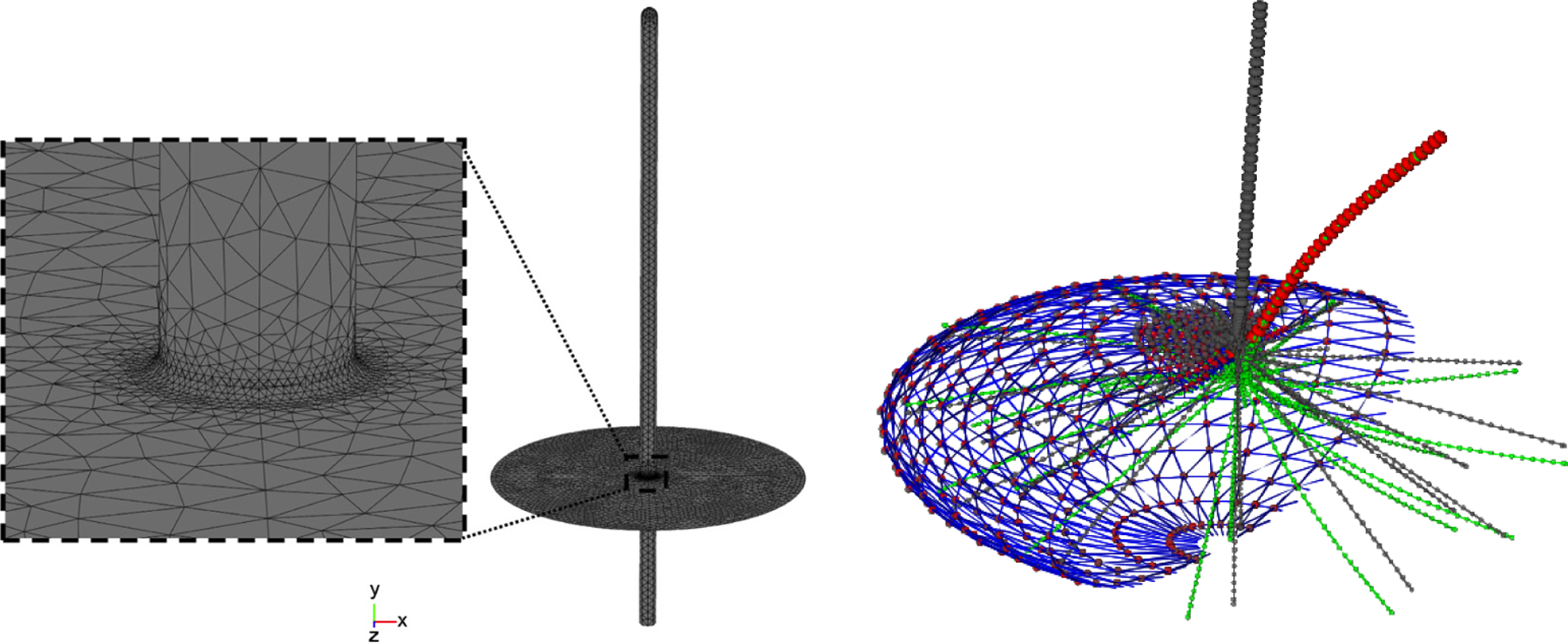
Continuum-based (left panel, reproduced from [50] with permission) and particle-based (right panel, reproduced from [90] with permission) simulations of the biomechanics of primary cilia.

Computational models of the primary cilia have been developed to investigate their biomechanics using various numerical methods, including low-dimensional and 3D models of the primary cilium discussed in the review article by Lim et al. [108]. Rydholm et al. developed the first 3D finite element method (FEM) model of the primary cilium to deduce the relation between cilium bending and membrane stress [50]. In their FEM study using COMSOL Multiphysics [109], the cilium is modeled as a continuous and homogeneous cylinder without basal body rotation, as shown in the left panel in Figure 4. They predicted the maximum strain at the cilium base, and explored the possibility of calcium response delay due to viscoelastic properties of the membrane by combing the modeling with experiments. Downs et al. then developed a computational model of the cilium accounting for both the base rotation and the initial shape of the cilium [102]. They found that the average flexural rigidity of primary cilia might be higher than previously reported by combining the cilium model with experiments and 3D flow simulations using COMSOL [109]. Mathieu et al. conducted FEM analysis of strain amplification caused by tensile strain applied to a primary cilium of a human adipose-derived stem cell (hASC) embedded in a collagen matrix rather than typical fluid shear loading [110]. Their results indicated that the cilium orientation might be more important than the cilium length in determining sensitivity of hASC to tensile strain.

In addition, Vaughan et al. developed fluid–structure interaction models to characterise the deformation of integrin- and primary cilia-based mechanosensors in bone cells under fluid flow stimulation [111]. They discovered that integrin attachments on the cell–substrate interface were highly stimulated, while the presence of a primary cilium on the cell also resulted in significant strain amplifications, arising at the ciliary base. Khayyeri et al. applied FEM to simulate an entire cell with multiple interacting mechanical components, including the primary cilium, actin and MT networks, and the nucleus [112]. They investigated how the mechanical properties of the primary cilium are involved in the mechanotransduction process whereby cilium deflection under fluid flow induces strains on the internal cell components that regulate the cell’s mechanosensitive response. Cui et al. applied a 2D immersed boundary-lattice Boltzmann method to investigate the dynamics of a primary cilium in pulsatile blood flows with two-way fluid–structure interaction [113]. They found that the location of the maximum tensile stress is not always found at the basal region and it is able to propagate from time to time within a certain distance to the base in the case of pulsatile flows.

Recently, Flaherty et al. [90] uncovered the anisotropic nature of the ciliary axoneme and applied the anisotropic shell model for the MTs [85] to understand the mechanics of a primary cilium under an optical trap where the thermal fluctuations of the cilium tip are experimentally measured. Their results showed that the primary cilium has a length-dependent bending rigidity (Figure 3D) and the active motion of the basal body contributes significantly to the tip fluctuations. Furthermore they also developed a dissipative particle dynamics (DPD) model to simulate the thermal fluctuations of the cilium with the rotation of the basal body and the deformation of the whole cell as shown in the right panel in Figure 4.

Despite these successful computational studies of primary cilia and continuously growing power of numerical simulations, there are still several major challenges in predicting the biomechanics of the primary cilia using numerical simulations. First, the cilium mechanics is a multiscale problem across vastly temporal and spatial scales [114], and it is a challenge to connect the biophysics of proteins such as polycystins and tubulins at the molecular scale to the response of the cilium and the whole cell at the cell scale, and even to the response of the tissue such as renal collecting tubules. While continuum-based FEM modeling were used in most existing computational studies of mechanics of primary cilia (left panel in Figure 4), to address the challenge of multiscale modeling, particle-based [90] (right panel in Figure 4) and atomistic-based modeling have to be integrated with the continuum-based modeling, as continuum-based modeling is not valid at the molecular scale. Second, the detailed ultrastructure of the primary cilium is still under refinement, which is critical for accurate modeling of the cilium. For example, recently serial section electron tomography showed that the cilium axoneme structure is relatively stable but gradually evolves from base to tip with a decreasing number of MT complexes and a reducing diameter [115]. In addition, actin structures were also discovered in the axoneme revealed by cryo-electron tomography [116]. Finally, as explored by Khayyeri et al. [112], the primary cilium is one component of the whole cell structure. To accurately capture the cilium response, other structural components connecting to the basal body, especially the actin cortex, cytoplasmic MT network, and striated rootlet, need to be modeled realistically as well.

### 4.2. Mechanotransduction of primary cilium

To understand the full function of the primary cilium both as a mechanosensor and its relevance to disease, it is also important to computationally predict the biochemical signalling pathways such as calcium dynamics besides the biomechanics of the primary cilium. The unique mechanosensation pathway of primary cilia related to polycystin channels has not been explicitly simulated using numerical methods, but several relevant mechanosensation pathways have been computationally investigated, which can be potentially modified to study the pathway in primary cilia. Although extensive studies have been done on mechanosensation of endothelial cells (ECs), especially related to vascular endothelial cells [117], there is little computational study on epithelial cell mechanotransductions. In the endothelial cells, the primary cilium is considered one of the ‘decentralized mechanisms’ to the mechanosensations, besides glycocalyx layer, focal adhesion, cytoskeleton, and cell-cell junctions [118]. For example, Sriram et al. developed a biochemical model of the wall shear stress-induced activation of endothelial nitric oxide synthase (eNOS) in an endothelial cell [119]. Barakat and co-workers studies the effect of flows on endothelial cell mechanotransduction [120–122], and investigated the relative contributions of shear stress and transport to nucleotide concentration at the EC surface. Ordinary differential equations (ODEs) are typically employed to solve the transport and reactions of signaling species using compartment models. These include extracellular compartment, cytoplasmic compartment, and nucleus compartment separated by plasma membrane and nuclear membranes. On the other hand, when the cellular geometry is available, partial differential equations (PDEs) can be solved to resolve the temporal and spatial distributions of specie concentrations, although more computationally expensive. In addition to endothelial cells, mechanotransduction of a hair cell in the inner ear has also been studied [123, 124], which is highly relevant to primary cilium mechanosensing. Besides, mechanosensation pathways in simpler red blood cells have also been investigated. For example, Zhang et al. applied a Lattice-Boltzmann model to predict the release of ATP due to mechanosensing of red blood cell and the concentration of ATP in the 3D space and how it evolves with time by solving the convection-diffusion-reaction PDEs [125].

**Figure 5.**
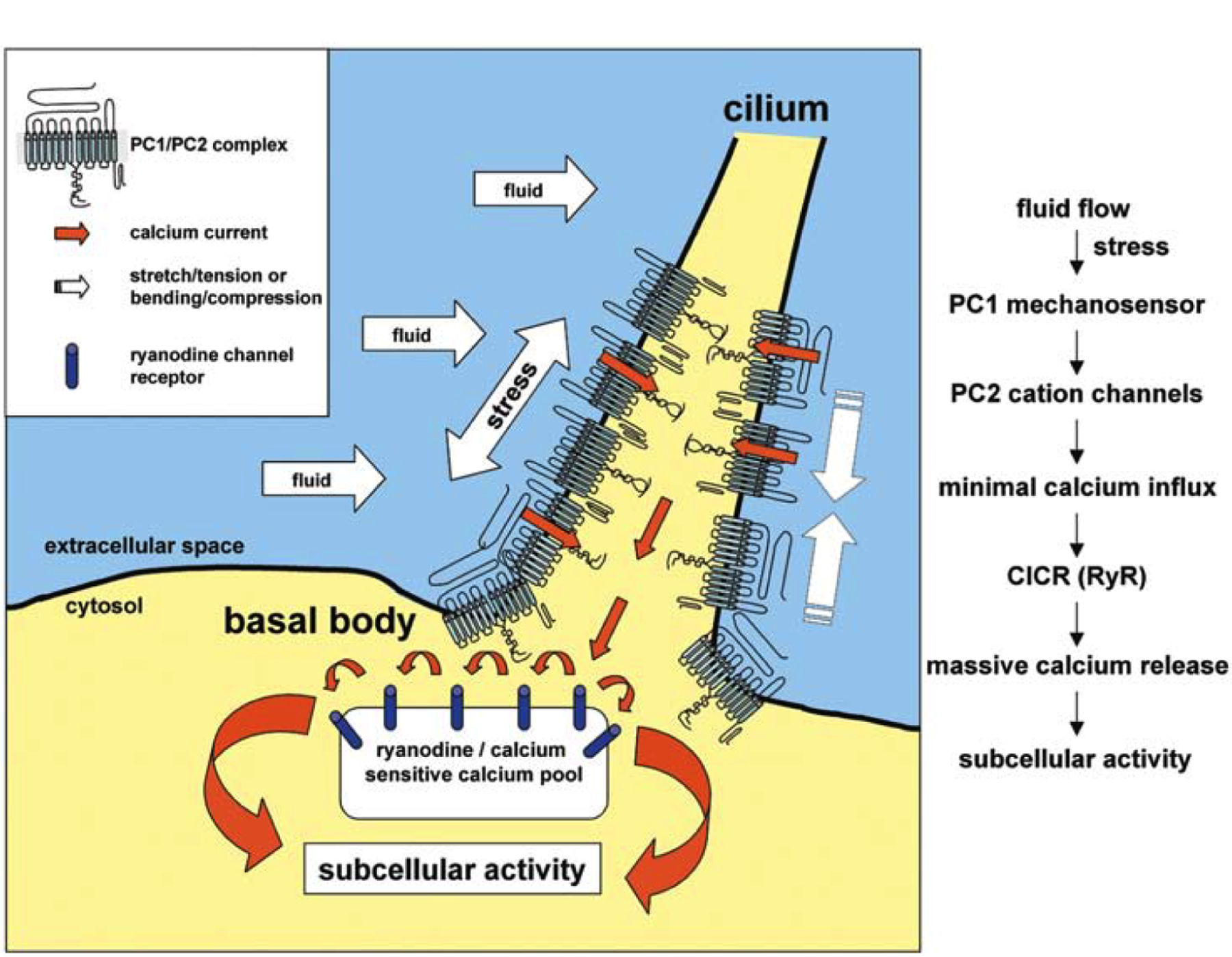
Schematic diagram of primary cilium mechanotransduction (reproduced from Nauli et al. [52] with permission).

Although many details need to be further confirmed experimentally, mechanosensing via primary cilia can be described as following [52], as shown in Figure 5. As the cilium bends under flow the fluid stress is first transmitted to polycystin-1 (PC1) that acts as a sensory molecule. Then conformational change of PC1 triggers opening of PC2. PC2 (a cation channel) then opens up and produces sufficient Ca^2+^ influx to activate intracellular channels such ryanodine receptors (RyRs) through calcium-induced-calcium release (CICR), which has been extensively studied for the cardiac myocytes in the heart [126–130]. In this case polycystin complex (PC) is either closed (C) or open (O), gated by the stress transduced from the cilium deflection due to fluid flow. In the pool of RyRs, each receptor can be open or closed depending on the amount of calcium from the opening of PC.

These Ca^2+^ dynamics have not only been studied theoretically [131,132], but also been investigated computationally [133–135]. Both continuum modeling approaches such as finite element analysis and discrete modeling approaches such as Monte Carlo method have been applied to solve the equations of Ca^2+^ dynamics. For example, Panday and Pardasani adopted finite element technique to portray calcium diffusion in oocytes in presence of sodium calcium exchanger [136]. Naik and Pardasani, Jha and Adlakha have used finite element method to delineate the calcium concentration distribution in various cells and the effect of RyR calcium channel, ER leak and SERCA pump [137, 138]. Naik and Pardasani proposed the following model to describe spatial and temporal Ca^2+^ concentration [Ca^2+^] in CICR in oocyte cells as [138]

(4.1) $$\frac{\partial \left[{\text{Ca}}^{2+}\right]}{\partial t}\;=\;D_{\text{Ca}}\nabla^{2}\left[{\text{Ca}}^{2+}\right]\;-\;k_{j}^{+}{\left[B_{j}\right]}_{\infty }\left(\left[{\text{Ca}}^{2+}\right]\;-\;{\left[{\text{Ca}}^{2+}\right]}_{\infty }\right)\;+\;V_{RyR}P_{o}\left({\left[{\text{Ca}}^{2+}\right]}_{ER}\;-\;\left[{\text{Ca}}^{2+}\right]\right)\;-\;V_{S\;\text{ERCA}}\left(\left[{\text{Ca}}^{2+}\right]\right)\;+\;V_{\text{Leak}}\left({\left[{\text{Ca}}^{2+}\right]}_{ER}\;-\;\left[{\text{Ca}}^{2+}\right]\right)\;+\;\delta \;\cdot \;\sigma_{\text{Ca}}$$

where the 1st term is related to diffusion, 2nd term related to endogenous Ca^2+^ buffers, 3rd term due to ryanodine receptors, the 4th term related to SERCA pumps, the 5th term related to leak of Ca^2+^ from ER membrane, and the last term related to a Ca^2+^ source, such as Ca^2+^ membrane channels. *D*_Ca_ is the di usivity of Ca^2+^ ions, $k_{j}^{+}$ is the association rate for jth buffer, [*Bj*]_∞_ is the buffer concentration at equilibrium and [Ca^2+^]_∞_ is the Ca^2+^ concentration far from the source. *V*_*RyR*_ is the ryanodine receptor rate, *P*_*o*_ is the open probability of RyR and [Ca^2+^]_*ER*_ is the ER Ca^2+^ concentration. *V*_*SERCA*_ is the maximum Ca^2+^ uptake through SERCA pump. The source term *σ*_Ca_ depends on the mechanical stress on the cilium axoneme [139]. The various reaction rates are often assumed to be constant, while mechanical stress is known to affect the reaction rates in mechanochemical processes [140,141].

The sarco/endoplasmic reticulum calcium ATPase (SERCA) is a membrane protein expressed in the ER of all cells [142]. SERCA2b is expressed in all cells including smooth muscle cells (SMC), endothelial cells (EC), and platelets. *V*_*Leak*_ is the leak conductance of Ca^2+^ from ER membrane. is the Dirac function representing the location of a Ca^2+^ source with the amplitude of *σ*_Ca_. This equation is then solved by a 3D finite element method in cylindrical geometry. It is reported that the temporal dynamics and spatial distribution of Ca^2+^ depends on the geometry of the Ca^2+^ influx [138]. This finding illustrates the importance to identify calcium influx along the cilium axoneme to determine the calcium responsiveness of the primary cilium under flow.

Besides continuum modeling approaches, Monte Carlo modeling approaches were also used to study Ca^2+^ dynamics. For example, Williams et al. applied a probability density approach and Monte Carlo calculations to predict localized Ca^2+^ influx via L-type Ca^2+^ channels and CICR release mediated by clusters of ryanodine receptors during excitation-contraction coupling in cardiac myocytes [130].

There are even more challenges in the simulation of mechanotransduction of the primary cilium than its biomechanics. First, it is still controversial whether the primary cilium is a calcium responser via polycystins, as Delling et al. showed that there is no calcium signal inside the cilium [143]. Second, the delay of calcium signal were confirmed in several experiments [50], but whether the delay is due to viscoelastic properties of the membrane or the calcium diffusion in the cilium and cell, or the CICR remains unknown. It is crucial to experimentally and computationally measure which process is the rate-limiting step in the calcium signal delay. Furthermore, how to validate the simulation of calcium dynamics by experiments is another challenge, especially for the 3D spatial and temporal distributions. Finally, besides calcium dynamics, there are other signalling pathways involved, including purinergic, ATP, nitric oxide (NO), reactive oxygen species (ROS), and mammalian target of rapamycin (mTOR) signalling pathways [144]. How to integrate these signalling pathways in the numerical simulations is a non-trivial task.

In addition, the coupling between the mechanical and chemical components of the primary cilium can be two-way. Not only the mechanical stress can induce chemical signals, but also the down stream signal pathways can impact the mechanical properties of the cilium, such as altering the cilium length. However, the time scale of the adjustment of cilium length due to mechanosensing might be much longer than that of the Ca^2+^ signalling [9]. Furthermore, it remains unknown whether coupled continuum approaches could be sufficient for capturing the coupling between the chemical and mechanical systems without resorting to atomistic approaches. For example, the effective medium continuum approach for diffusion [145] might not be accurate enough to capture the diffusion of Ca^2+^ through the cytoskeletal network with its own distributed charges, and atomistic-based approach or coarse-grained particle simulations should be more realistic.

## 5. Open questions

Understanding primary cilium function represents a canonical multiscale modeling problem, spanning multiple length scales and time scales. Research efforts directed at the primary cilium and its physiological role live at the intersection of a variety of fields, ranging from the microscale (particle dynamics, molecular imaging) through the mesoscale (cellular physiology) and the macroscale (tissue and organ fluid dynamics, homeostasis). Development of multiscale models in general face a variety of difficult conceptual problems [146] and we hope that studies of primary cilia gain increased attention as a model system to develop multiscale models.

Primary cilia could be considered as a simple model of ‘active matter’, as kinetic energy from moving fluid is converted into signalling activation that drives systemic biochemical responses. The primary cilium structure itself is passive, meaning that primary cilia do not generate motion but only respond to imposed forces, and the mechanical response is overdamped. However, energy transduction processes occur that “somehow” first converts kinetic energy of moving fluid into mechanical strain energy and finally into biochemical potential energy (signalling pathways) that results in altered cell and tissue function. This transduction process is not currently understood but there is experimental evidence supporting the connection between pathological cilium length and organ pathophysiology, for example [77].

In any case, there remains several important open problems: (I) In terms of structural models, a principal question is “given new and improved microstructural observations of primary cilia, are existing models sufficient to describe ciliary mechanics, or do they need to be modified”? Conversely, this question could be posed as “given differing molecular-scale structural models of a cilium, what meso- or macroscale experiments can distinguish between competing models”? To use a specific example from [115] two obvious follow-up questions arise. First, are the variations of length of individual axonemal MTs that were observed in one cilium identical to, or representative of, in vivo cilia? Second, are the reported axonemal MT length variations constant, or are axonemal MTs subject to stochastic length variations? (II) In terms of functional models, relating cilium structure to cilium function is perhaps the central question. Definitive Identification of the initial site of ciliary mechanotransduction and determination of flow-sensing sensitivity are the necessary first steps here.

Other questions readily present themselves: why do cells sense fluid flow? How do cells respond to fluid flow? How does a cell-level flow sensor output become integrated into organ or organism-level homeostatic regulation? One known example is how applied fluid flow interacts with the Planar Cell Polarity signalling pathway to control (spatial) tissue growth [147]: tubules grow longer, not wider, by flow-regulated orientation of the mitotic spindle. Primary cilia are an excellent model system for exploring how information about the physical external environment is transduced into internal chemical information (activation of signalling pathways, altered gene expressions, altered cell and tissue function, and homeostatic mechanisms). We emphasize that experimental data on this point is currently sparse but growing.

There is an important consideration facing modelers: there is no such thing as a generic ‘cell’ or even a generic ‘mammalian epithelial cell’. Epithelial tissue is formed from all three of the primary germ layers: ectoderm, mesoderm, and endoderm, yet ectodermal-derived retinal pigment epithelia are clearly significantly different in form and function from mesodermal-derived kidney epithelia. Models of the structure and function of primary cilia must account for the manifold physiological differences between cell types. Similarly, it must be noted that “terminally differentiated” cells [148] expressing cilia have significant differences in both gene expression and physiology compared to cells trapped in the cell cycle- differences often not fully appreciated.
